# Advanced SnO_2_ Thin Films: Stability and Sensitivity in CO Detection

**DOI:** 10.3390/ijms252312818

**Published:** 2024-11-28

**Authors:** Nadezhda K. Maksimova, Tatiana D. Malinovskaya, Valentina V. Zhek, Nadezhda V. Sergeychenko, Evgeniy V. Chernikov, Denis V. Sokolov, Aleksandra V. Koroleva, Vitaly S. Sobolev, Petr M. Korusenko

**Affiliations:** 1 Laboratory of Optical Materials and Coatings, National Research Tomsk State University, Tomsk 634050, Russia; nkmax3@yandex.ru (N.K.M.);; 2 Laboratory of Semiconductor Devices, National Research Tomsk State University, Tomsk 634050, Russia; 3 Laboratory of Metal Oxide Semiconductors, National Research Tomsk State University, Tomsk 634050, Russia; 4 Department of Physics of Nanomaterials and Heterostructures, Omsk Scientific Centre of the Siberian Branch of the Russian Academy of Science, Omsk 644013, Russia; 5 Research Park, Saint Petersburg State University, Saint Petersburg 199034, Russia; 6 Department of Solid-State Chemistry, Saint Petersburg State University, Saint Petersburg 199034, Russia; 7 Department of Physics, Omsk State Technical University, Omsk 644050, Russia

**Keywords:** carbon monoxide (CO) sensor, thin film, tin dioxide, silver, rare earth element (Dy), stability

## Abstract

This paper presents the results of a study on the characteristics of semiconductor sensors based on thin SnO_2_ films modified with antimony, dysprosium, and silver impurities and dispersed double Pt/Pd catalysts deposited on the surface to detect carbon monoxide (CO). An original technology was developed, and ceramic targets were made from powders of Sn-Sb-O, Sn–Sb-Dy–O, and Sn–Sb-Dy-Ag–O systems synthesized by the sol–gel method. Films of complex composition were obtained by RF magnetron sputtering of the corresponding targets, followed by technological annealing at various temperatures. The morphology of the films, the elemental and chemical composition, and the electrical and gas-sensitive properties were studied. Special attention was paid to the effect of the film composition on the stability of sensor parameters during long-term tests under the influence of CO. It was found that different combinations of concentrations of antimony, dysprosium, and silver had a significant effect on the size and distribution of nanocrystallites, the porosity, and the defects of films. The mechanisms of degradation under prolonged exposure to CO were examined. It was established that Pt/Pd/SnO_2_:0.5 at.% Sb film with optimal crystallite sizes and reduced porosity provided increased stability of carbon monoxide sensor parameters, and the response to the action of 100 ppm carbon monoxide was *G*_1_/*G*_0_ = 2–2.5.

## 1. Introduction

For mobile monitoring of various gaseous media, an urgent task is the development of miniature sensors based on metal oxide semiconductors, characterized by low energy consumption, high performance, and low cost. At the same time, an important and often still unresolved problem is the study of the mechanisms of degradation of sensor parameters and the search for ways to ensure their stability during long-term operation. The reviews [1,2] considered engineering approaches used to improve sensitivity and selectivity, as well as the stability and reliability of semiconductor gas sensors. At the same time, it was noted that these methods cannot replace traditional approaches based on the search for new materials and the development of new technologies that improve and optimize gas-sensitive layers. The most promising method remains chemical modification of the surface and volume of the sensor material [3,4,5,6,7]. In the review [8], the authors summarized the results of their studies on the active centers and gas-sensitive behavior of n-type semiconductor metal oxides with different compositions (simple oxides ZnO, In_2_O_3_, SnO_2_, and WO_3_; mixed metal oxides BaSnO_3_ and Bi_2_WO_6_) functionalized with catalytic noble metals (Ru, Pd, and Au). It is important to note that this work is devoted to semiconductor materials obtained by thick-film technology. In addition, this review considers the composition, metal–oxygen bonds, microstructure, active centers, sensory behavior, and interaction pathways of these materials with gases (CO, NH_3_, SO_2_, volatile organic compounds, and NO_2_). As a result, the key role of active centers in determining the selectivity of sensor materials is substantiated. At the same time, one of the main problems remains the stability of catalytic additives under sensor operating conditions.

In order to develop new scientific approaches to the creation of sensors with given parameters, it is necessary to conduct a comprehensive study of the relationship between the manufacturing technology, composition, microstructure, and properties of metal–oxide semiconductors with various catalytic additives. In the studies [9,10,11,12], the degradation mechanisms were studied, and the ways of increasing the stability of sensors for a number of gases based on thin (about 100 nm) tin dioxide films with dispersed Pt, Pd, and Au catalysts on the surface and various impurities (Pt, Ni, Co, Au, Ag, Y, and Sc) in the bulk of the films were identified. It is important to note that thin films were formed by direct current magnetron sputtering of mosaic metal targets made of Sn + Sb alloy with corresponding metals deposited on the surface.

The most difficult task is to ensure long-term stability of the parameters of carbon monoxide sensors [13,14,15]. To create trace CO sensors with reduced energy consumption and stable parameters, it is advisable to use thin-film Pt/Pd/SnO_2_:Sb sensors in a thermocycling mode (heating to 400 °C for ≥2 s and cooling to 70 °C for ≥5 s) subjected to technological tests for 7–8 days [16]. However, such conditions are not always appropriate for practical developments when it is necessary to simultaneously detect several gases using different sensors. Industrial production of gas sensors requires the development of a technology that includes magnetron sputtering of targets of complex composition to obtain thin-film sensors with additives of noble, 3*d*, and rare earth metals. In combination with microelectronic technology, it is possible to obtain a large number of miniature sensitive elements with identical characteristics in one technological cycle. The authors of this study conducted studies aimed at creating original oxide ceramic targets of various compositions from powders synthesized by the sol–gel method [17]. As a result, modes of RF magnetron sputtering of targets for the formation of thin-film sensors were developed.

In this work, we studied the elemental and chemical composition as well as the structural, electrophysical, and gas-sensitive properties of thin (about 150 nm) SnO_2_ films obtained by RF magnetron sputtering using original technology ceramic targets from powders of the Sn-Sb–O, Sn-Sb–Dy–O, and Sn-Sb-Dy–Ag-O systems, with dispersed Pt/Pd catalysts applied to the surface in order to create carbon monoxide sensors with increased stability during operation.

## 2. Results and Discussion

### 2.1. AFM

**Figure 1:**
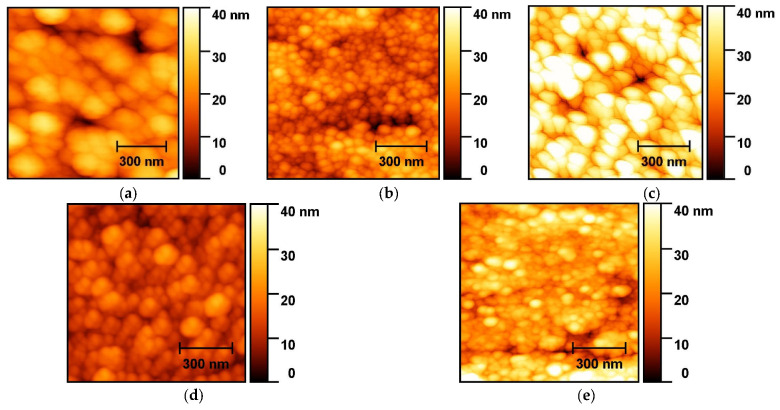
AFM images of samples: series (I)-693K (**a**), series (II)-693K (**b**), (III)-723K (**c**), series (IV)-693K (**d**), and (V)-723K (**e**).

Figure 1 shows AFM images of the studied samples. Analysis of the AFM images allowed us to establish that the surface of the films had a granular structure with grains that were close to spherical in shape and an average radius in the range of 18–63 nm (Figure 1, Table 1). As can be clearly seen in Figure 1a, the film of the (I)-693K series had an average grain size of 63.1 ± 8.6 nm, and its roughness and porosity were 7.4 ± 1.9 nm and 19.3 ± 2.4%, respectively. For the film of the (II)-693K series (Figure 1b), an increase in porosity by 1.6 times and an insignificant decrease in its roughness were observed compared to the sample of the (I)-693K series. This result was most likely associated with a decrease in the average grain size by 1.8 times. It should be noted that from the literature [18], it is known that the introduction of rare earth elements contributes to a decrease in grain size in metal oxide semiconductors. In the case of a sample of the same series, but after annealing at 723 K ((III)-723K), the statistical parameters of the surface changed in a certain way, namely, the average grain size increased from 34.9 ± 13.8 to 50.7 ± 15.0 nm (Figure 1c). As a result, the porosity decreased and the surface roughness of this film increased. It is interesting that the surface morphology of the films of the (IV)-693K and (II)-693K series (Figure 1b,d) was visually somewhat different, although all the surface parameters determined from the AFM images were close within the spread of average values. At the same time, when moving from the (IV)-693K series sample to the (V)-723K sample (Figure 1d,e), an insignificant decrease in porosity was observed despite the fact that the roughness increased almost 2 times with a simultaneous decrease in the grain size by 1.7 times. This result may be due to the fact that at a higher technological annealing temperature, the initially spherical grains begin to stretch in height (perpendicular to the film) with a simultaneous narrowing of their radial dimensions in the film plane.

Thus, annealing of the films of the (II)-693K and (IV)-693K series at 693 K led to an increase in their porosity by 1.4–1.6 times due to a decrease in the average crystallite size and roughness by 1.8–2 times compared to the film of the (I)-693K series (Table 1). At the same time, the addition of Ag did not result in noticeable changes in the surface topography of the films. However, in the case of annealing at 723 K, the presence of Ag radically affected the morphology of the film, with the exception of porosity, which changed slightly. In other words, the addition of silver and technological annealing at 723 K led to a decrease in the grain size relative to the film of the (I)-693K series by more than three times, despite an insignificant decrease in roughness. It is also important to note that in the films of the (V) series with Dy + Ag, the spread in grain sizes was the smallest (Table 1), which indicates greater self-organization of crystallites.

### 2.2. XPS

**Figure 2:**
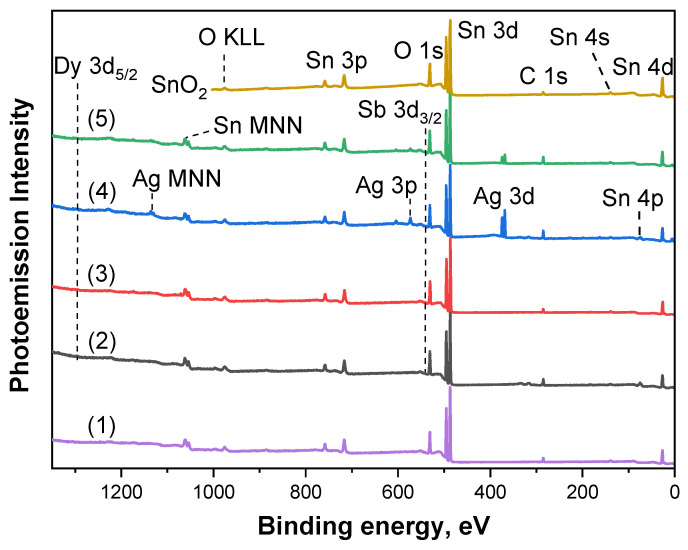
Survey PE spectra of samples: (1)—(I)-693K, (2)—(II)-693K, (3)—(III)-723K, (4)—(IV)-693K, (5)—(V)-723K, and reference SnO_2_.

The chemical composition of the studied samples was determined by analyzing individual core PE lines of elements in the survey spectra (Figure 2), taking into account the known atomic sensitivity factors [19]; all data are summarized in Table 2. In all survey spectra, PE lines of tin (Sn 3*p*, Sn 3*d*, Sn 4*s*, Sn 4*p*, and Sn 4*d*), oxygen (O 1*s*), and carbon (C 1*s*), as well as Auger lines of Sn MNN and O KLL, were observed. The presence of carbon on the surface of the samples was probably due to its adsorption from the atmosphere. In the case of films (II)-693K, (III)-723K, (IV)-693K, and (V)-723K, in addition to the above-mentioned PE lines, the presence of dysprosium Dy 3*d* and antimony Sb 3*d*_3/2_ lines was also observed, and in samples (IV)-693K and (V)-723K, PE lines of silver (Ag 3*p* and Ag 3*d*) and Auger lines of Ag MNN were also present. No other foreign elements were found in the studied films. It can be clearly seen from the data in Table 2 that the concentration of the main elements, tin and oxygen, for all samples was in the range of 22.6–28.2 at.% and 65–74.5 at.%, respectively. The concentration of the dopant antimony was approximately the same for samples (II)-693K, (III)-723K, (IV)-693K, and (V)-723K and was 0.31–0.36 at.%; in the case of sample (I)-693K, Sb was not detected. The main reason for its absence in the latter sample is probably related to the fact that the real concentration of antimony is below the detection limit for this element, as the declared concentration of Sb in this sample is two times lower than for the other samples of SnO_2_ films. At the same time, the concentration of dysprosium, an active impurity in the samples (II)-693K, (III)-723K, (IV)-693K, and (V)-723K, changed slightly when passing from the (II)-693K and (III)-723K series to the (IV)-693K and (V)-723K series, namely, for the (II)/(III) series, the Dy concentration was 0.5–0.6 at.%, and for the (IV)/(V) series, it was about 0.4 at.%. In addition, for the (IV)-693K and (V)-723K series, in addition to Sb, silver and dysprosium were also introduced into the composition of the films at the stage of their formation. It is interesting that for the samples of this series, the annealing temperature of the film significantly affected the Ag concentration. Thus, during annealing at 693 K, the silver concentration was 11.6 at.%, while at a higher technological annealing temperature of 723 K, the concentration decreased to ~4 at.%. In other words, the technological annealing temperature plays an important role in the distribution of silver, which is localized either on the surface or in the bulk of the SnO_2_ film. It is also important to note that the ratio of the concentration of [Sn] to [O] was 2.85–3.0 depending on the sample, i.e., there was an oxygen surplus. This result suggests that some of the oxygen is probably chemically bound to carbon.

**Figure 3:**
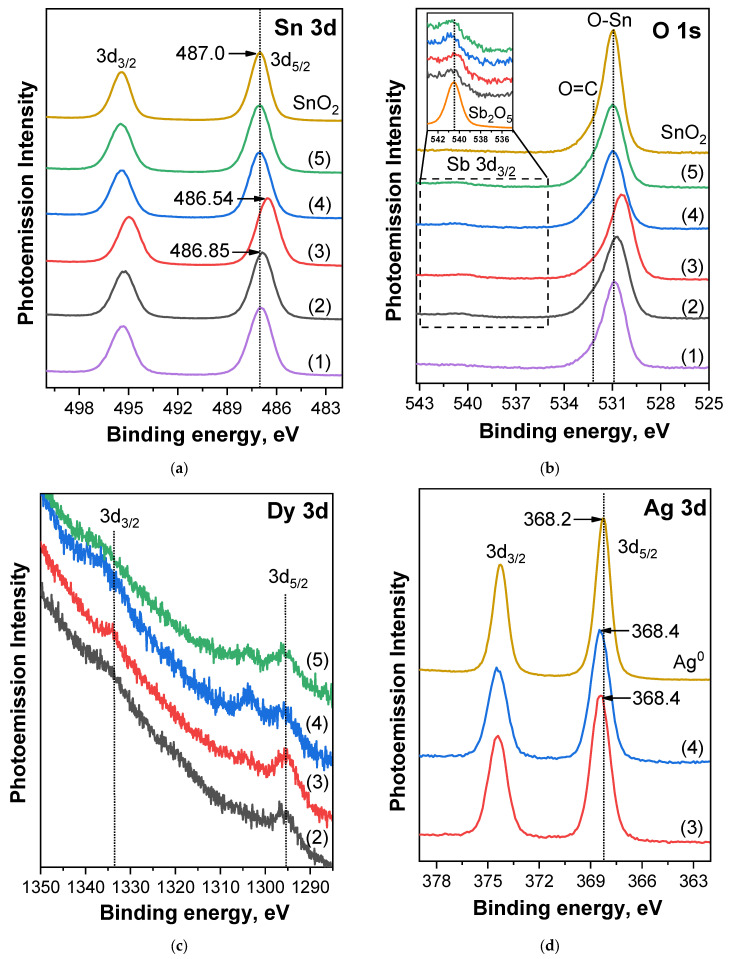
Sn 3*d* (**a**), O 1*s* with Sb 3*d*_3/2_ (**b**), Dy 3*d* (**c**), and Ag 3*d* (**d**) PE spectra of samples: (1)—(I)-693K, (2)—(II)-693K, (3)—(III)-723K, (4)—(IV)-693K, (5)—(V)-723K, and reference compounds (SnO_2_, Sb_2_O_5_, and Ag^0^).

For a detailed analysis of the chemical state of the elements in the composition of thin SnO_2_ films, let us consider Figure 3, which shows Sn 3*d*, O 1*s*, Sb 3*d*_3/2_, Dy 3*d*, and Ag 3*d* PE spectra.

Let us start with the consideration of the Sn 3*d* PE spectra of the studied samples (curves 1–5 in Figure 3a), which consisted of 3*d*_5/2_ and 3*d*_3/2_ two spin-orbit doublets. The energy distance between the doublet lines for all samples was 8.4 eV, which coincided with a similar value of the distance for the reference compound SnO_2_ (brown curve in Figure 3a). In addition, the most intense 3*d*_5/2_ line (which will be further considered as the main one) for the studied samples had the same full width at half maximum (FWHM) as SnO_2_. All this indicates that the tin in all the films was in the same chemical state in the form of the higher oxide SnO_2_. A detailed comparison of the spectra of thin films with the spectrum of the reference compound SnO_2_ showed that the main differences were observed in the low-energy shift of the Sn 3*d*_5/2_ line, which was manifested only for two samples: (II)-693K (−0.15 eV) and (III)-723K (−0.46 eV). For other samples, the position of this line coincided with the position for SnO_2_—487.0 eV (see Table 2). Interestingly, in the samples for which a low-energy shift was observed, dysprosium was added to antimony, and it was for them that higher concentrations of Dy were found. Moreover, for the sample (III)-723K, for which the strongest energy shift was also found, the concentration of Dy was the highest. Thus, the detected shift of the tin 3*d*_5/2_ line for samples (II)-693K and (III)-723K was associated precisely with the influence of the rare earth element dysprosium.

Now let us consider Figure 3b, which shows the O 1*s* spectra of the samples under study. As can be clearly seen (Figure 3b, Table 2), in general, the position of the O 1*s* spectra maximum for the (I)-693K, (IV)-693K, and (V)-723K films coincided with the position for the reference compound SnO_2_ (~530.9 eV). At the same time, a slight asymmetry of the O 1*s* lines on the side of high binding energies was also detected in all spectra, associated with the presence of C=O bonds from carbon on the surface of the films [20]. Thus, it can be concluded that oxygen in the samples under study was mainly in the lattice form due to tin. Interestingly, a low-energy shift of 0.2 and 0.5 eV relative to SnO_2_ was detected for the (II)-693K and (III)-723K samples, respectively. A similar shift with close binding energy values for the same samples was observed in the analysis of the Sn 3*d* spectra. It is important to note that in the case of samples (IV)-693K and (V)-723K, low-energy shifts for the Sn 3*d* and O 1*s* spectra were not detected. Analyzing the obtained results, it can be assumed that the cause of the shifts of the Sn 3*d* and O 1*s* spectra for samples (II)-693K and (III)-723K was the addition of dysprosium, which contributed to a change in the Sn-O bond energy.

The inset to Figure 3b shows an enlarged region of 536–543 eV, in which the antimony line Sb 3*d*_3/2_ can be observed. At the same time, the more intense 3*d*_5/2_ line overlaps the O 1*s* spectrum, which does not allow it to be used for analysis. In view of this, the main analysis was carried out further using the Sb 3*d*_3/2_ line. As evident, the low-intensity Sb 3*d*_3/2_ line was observed for all samples except for sample (I)-693K, for which the declared concentration was two times lower than for the other samples. Probably, the actual antimony concentration for this sample was below the detection limit, which is why Sb could not be detected. For samples (II)-693K, (III)-723K, (IV)-693K, and (V)-723K, pentavalent antimony replaced tetravalent tin atoms, led to donor impurity, and helped to reduce the resistance of gas sensors. However, for the samples (IV)-693K and (V)-723K, the energy shift of the Sb 3*d*_3/2_ line was +0.2 eV, while for (II)-693K and (III)-723K, it was 0 and −0.3 eV, respectively. Such a shift in direction may indicate an additional influence of other additives in the SnO_2_ films. Thus, in the case of the samples (II)-693K and (III)-723K, dysprosium was introduced, and in (IV)-693K and (V)-723K, silver was introduced in addition to dysprosium.

Now let us move on to examining Figure 3c, which shows the Dy 3*d* spectra for samples (II)-693K, (III)-723K, (IV)-693K, and (V)-723K. Below, we will consider only the most intense 3*d*_5/2_ line. It is clearly seen that the Dy 3*d*_5/2_ spectra are quite noisy, which complicates their interpretation. However, the position of the maximum of the 3*d*_5/2_ spectra for all samples was approximately the same: 1295.6 eV. According to the literature, the position of Dy 3*d*_5/2_ for metallic Dy and Dy_2_O_3_ is 1293.3 and 1296.5 eV, respectively [21,22]. Comparing these literature data with the results obtained in this work for SnO_2_ films with the addition of Dy, it can be concluded that dysprosium was in the form of Dy^3+^ cation in the crystal lattice of tin oxide in all samples. According to the data [23], rare earth elements are characterized by higher energies of breaking bonds with oxygen compared to tin: ΔH°_298_ = 171 kcal/mol for Y–O, ΔH°_298_ = 161 kcal/mol for Sc–O, ΔH°_298_ = 144 kcal/mol for Dy–O, and ΔH°_298_ = 127 kcal/mol for Sn–O. At the same time, it has been shown [9] that in thin films of tin dioxide obtained by magnetron sputtering, during heat treatment, yttrium and scandium atoms segregate on the surface of microcrystals, form strong bonds with lattice oxygen, and are present in the form of Y^3+^ and Sc^3+^ ions. Similar phenomena apparently occur when modifying tin dioxide with dysprosium. Moreover, according to the literature [24,25], rare earth impurities are used in thick-film gas sensors to reduce the size of microcrystals.

Finally, let us consider the Ag 3*d* spectra for the (IV)-693K and (V)-723K samples, shown in Figure 3d. As can be seen, these spectra contained two spin doublets, 3*d*_5/2_ and 3*d*_3/2_, and the distance between them was 6 eV. Comparing these results with the data for the reference compound Ag^0^ (brown curve in Figure 3d), it can be argued that silver in the (IV)-693K and (V)-723K films was in one single chemical state—metallic. The main difference between the Ag 3*d* spectra of the studied samples and the reference compound was associated with only a small shift of +0.2 eV, observed for both SnO_2_ films with Dy and Ag additives. It can be assumed that this shift is due to the peculiarities of the interaction between tin atoms in SnO_2_, as well as silver and dysprosium, and charge redistribution in these films.

### 2.3. Raman Scattering

For bulk SnO_2_ crystals with the cassiterite structure, the following modes are active in the Raman spectra: *E*_g_ at 476 cm^−1^, *A*_1*g*_ at 638 cm^−1^, *B*_2*g*_ at 782 cm^−1^, and *B*_1*g*_ at 123 cm^−1^, with the *A*_1*g*_ mode being the most intense [26]. Raman spectra for bulk materials and polycrystalline samples, where the particle size (grains, agglomerates, clusters, etc.) lies in the nanometer range, vary greatly, and the classical modes *A_g_*, *E_g_*, and *B*_1*g*_ shift and expand [27]. Doping with impurities can lead to the appearance of additional peaks and a change in the shape of the Raman spectrum.

**Figure 4:**
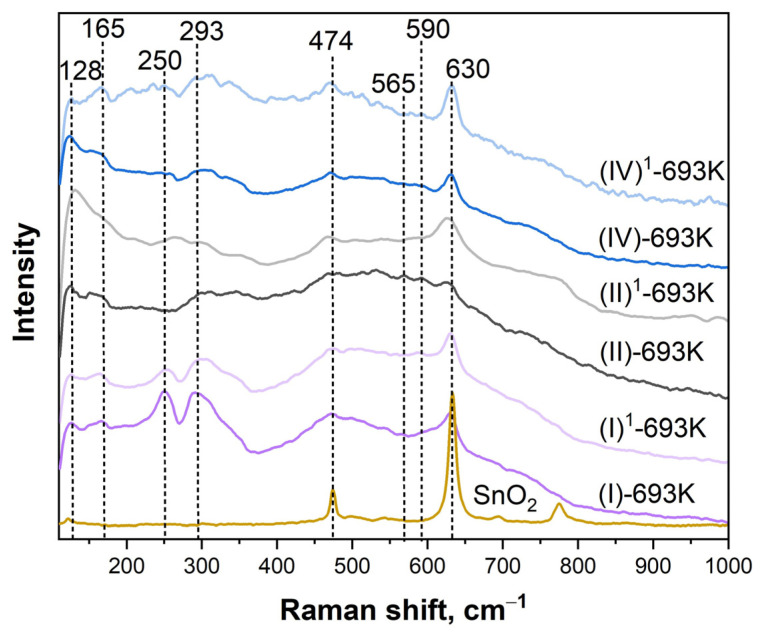
Raman spectra of SnO_2_ powder as well as samples (I)-693K, (II)-693K, and (IV)-693K before and after long-term (90 days) testing under CO exposure (designated by the number 1 superscript in the sample name).

In our case (Figure 4), the Raman spectrum of SnO_2_ powder was practically no different from coarse-crystalline samples [26]. When studying thin-film sensor structures, the main attention was paid to the samples that underwent technological annealing at 693 K, as preliminary studies showed that the parameters of these samples are of greater interest for increasing stability. In view of this, the spectra of freshly prepared sensors (I)-693K, (II)-693K, and (IV)-693K, as well as samples (I)^1^-693K, (II)^1^-693K, (IV)^1^-693K, were measured after long-term testing for 90 days under the influence of CO. For all nanocrystalline films modified with Sb, Dy, and Ag, the Raman spectra contained broad bands in the range of 100–800 cm^−1^ with a number of maxima, which is consistent with those observed in many nanostructured metal oxide films and is associated with size effects [27]. It is important to note that the *E_g_* (474 cm^−1^) and *A*_1*g*_ (630 cm^−1^) modes, characteristic of tin dioxide with a rutile structure, were also present in all spectra of the samples. However, in place of the *B*_1*g*_ mode, dual modes at 128 and 165 cm^−1^ were observed for all samples.

In the (I)-693K and (I)^1^-693K samples, bands with maxima at 250 and 293 cm^−1^ were clearly observed in the region from 200 to 350 cm^−1^, which are usually classified as anomalous and associated with infrared active *E*_u_ transverse optical and *E*_u_ longitudinal optical modes [26]. A feature of the Raman spectra of all the studied films was the presence of faint bands at 565 and 590 cm^−1^ in the high-frequency region. Previously [10], we showed that the Raman spectra of tin dioxide thin films with dispersed Pt/Pd catalytic layers deposited on the surface also contained bands with maxima at 565 and 590 cm^−1^. A detailed analysis of the experimental data showed that there were two states of platinum in tin dioxide: three-dimensional particles of metallic Pt^0^, which was inactive in Raman spectra, and the intermediate oxide PtO. This oxide was identified as two-dimensional dispersed platinum in the Pt^2+^ state and was responsible for the appearance of the band at 590 cm^−1^. The second band with a maximum at 565 cm^−1^ corresponded to a similar state of Pd^2+^. Thus, it can be concluded that long-term testing of the (I)-693K sample in a CO atmosphere did not change the state of the Pt/Pd bilayer catalysts. The introduction of dysprosium and silver impurities into the bulk of tin dioxide had a significant effect on the intensity and shape of the spectra of the freshly prepared (II)-693K and (IV)-693K samples, especially in the 200–400 cm^−1^ region. This result is consistent with the change in the microstructure of the (II)-693K and (IV)-693K films compared to the (I)-693K sample, as revealed from the AFM data (Figure 1, Table 1). It is important to note that the Raman spectra of samples doped only with antimony impurity were practically identical for freshly prepared (I)-693K and (I)^1^-693K that had undergone long-term testing under the influence of CO. However, the spectra of films with additives of dysprosium and silver before tests (II)-693K and (IV)-693K and after long-term tests (II)^1^-693K and (IV)^1^-693K changed significantly, which indicates the influence of carbon monoxide adsorption on the defectiveness of films.

### 2.4. Characterization of the Sensors

Adsorption of CO molecules on the surface of tin dioxide in air is accompanied by their oxidation due to previously chemisorbed oxygen:

CO + O^−^ → CO_2_↑ + e^−^.

As a result of this process, CO_2_ molecules are desorbed from the surface, and the electrons released during this process return to the conduction band, which leads to an increase in the conductivity of the sensor. In a real humid atmosphere, carbon monoxide can also interact with OH groups:

CO + 2OH^−^ → CO_2_↑ + H_2_O + e^−^.

However, with long-term exposure to CO, a decrease in response usually occurs, while the mechanism of degradation of sensor parameters remains unclear.

Analysis of previously obtained experimental data [9,10,12] shows that the change in the characteristics of Pt/Pd/SnO_2_:Sb sensors obtained by direct current (DC) magnetron sputtering of a Sn + Sb alloy target during long-term tests under the influence of CO is directly opposite to the patterns observed when the same samples are exposed to hydrogen. Due to the reaction of *H* atoms with oxygen released to the surface of microcrystals, the surface SnO_2_ molecules are reduced to Sn, which are the centers of oxygen chemisorption in the atomic form of O^−^. As a result, the band bending and response values increase. To prevent the reduction of tin dioxide and stabilize the parameters of hydrogen sensors, a combined introduction of Y + Ag or Pt impurities into the bulk of films is used [9,10]. However, large CO molecules are adsorbed primarily on the Pt/Pd catalyst particles, and due to the spillover effect, they reach the surface of the semiconductor and interact with pre-chemisorbed oxygen without penetrating the grain boundaries.

**Figure 5:**
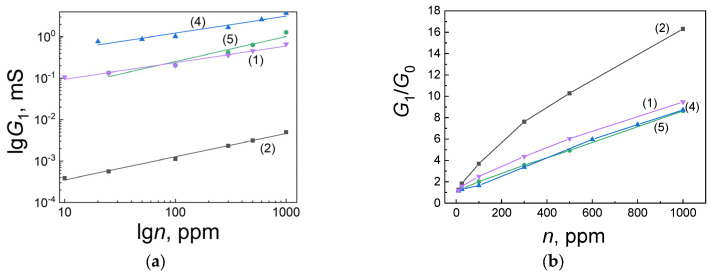
Graphs of conductivity versus (**a**) CO concentration and (**b**) response of freshly prepared sensors of series: (1)—(I)-693K, (2)—(II)-693K, (4)—(IV)-693K, and (5)—(V)-723K.

Figure 5 shows the concentration dependences of conductivity (a) and *G*_1_/*G*_0_ response (b) to the effect of carbon monoxide on freshly prepared sensors of the studied series. Particular attention was paid to the properties of films with a technological annealing temperature of 693 K because, according to preliminary data, such sensors are distinguished by more stable parameters.

For all sensors, the dependences of the conductivity *G*_1_ on the concentration of carbon monoxide in the range of 10–1000 ppm (Figure 5a) corresponded to the power law *G*_1_ = *α n m*, lg *G*_1_ = lg *α* + *m* lg *n* at the parameter values *m* = 0.4–0.6. Consequently, it can be concluded that the predominant role is played by the channel component of conductivity, which is realized in the presence of microcrystals that are connected to each other by narrow conductivity channels consisting of the same substance. The role of channels can be played by small nanocrystals in the grain boundaries, which large CO molecules do not penetrate. In general, analyzing Figure 5, it can be noted that the values of conductivity and response to CO depend on the composition of the films and the temperature of the technological annealing.

**Figure 6:**
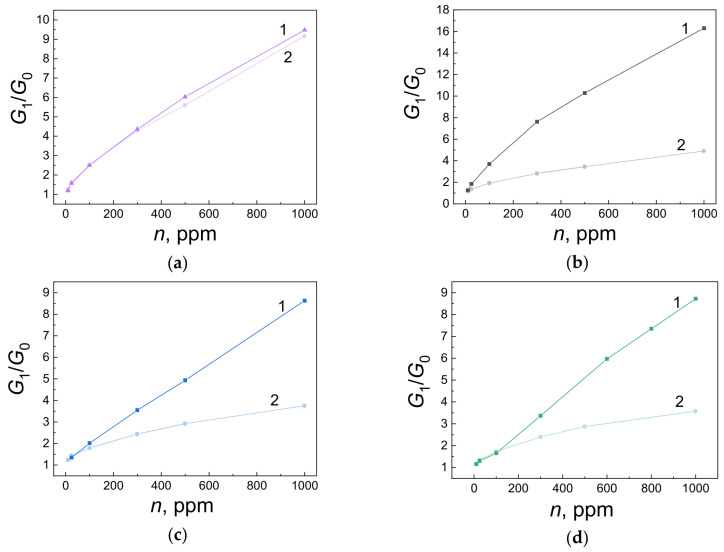
Concentration dependences of the response of freshly prepared sensors (curves 1) and sensors after long-term (90 days) testing (curves 2). Films from different series are presented: (**a**)—(I)-693K, (**b**)—(II)-693K, (**c**)—(IV)-693K, and (**d**)—(V)-723K.

Figure 6 shows the concentration dependences of the response for freshly prepared sensors (curves 1) and those that have undergone long-term (90 days) testing under periodic exposure to carbon monoxide in the operating mode at 673 K (curves 2). Table 3 summarizes all the main parameters of the sensors.

Let us consider the influence of Sb, Dy, and Ag additives in the SnO_2_ bulk and the annealing temperature on the microstructure and defects of the films as well as on the characteristics and stability of the sensor parameters.

According to the data obtained by AFM and XPS methods, the film of the (I)-693K series (Pt/Pd/SnO_2_: 0.5 at.% Sb) had the largest grain size of 63.1 ± 8.6 nm, and its roughness and porosity were 7.4 ± 1.9 nm, and 19.3 ± 2.4%, respectively (Figure 1, Table 1). The chemical composition of this sample corresponded to SnO_2_, and 0.5 at.%, antimony was not detected by the XPS method. The Raman spectra (Figure 4) showed the main bands characteristic of SnO_2_ with a rutile structure. At the same time, the presence of broad bands in the region of 100–800 cm^−1^ is consistent with those observed in many nanostructured metal oxide films and is associated with size effects. For this sample, the value of the energy band bending at grain boundaries *eφ_s_* did not exceed 0.22–0.25 eV, and the response to the action of 100 ppm was *G*_1_/*G*_0_ = 2–2.5 (Table 3, Figure 5b). It is important to note that during tests in the constant heating mode, the Raman spectra remained virtually unchanged (curves (I)-693K and (I)^1^-693K in Figure 4), and the sensor parameters remained stable (Figure 6a and Table 3).

In the films of the (II)-693K series, during technological annealing at 693 K, dysprosium (0.5–0.6 at.%) was released on the surface of tin dioxide microcrystals and formed a strong bond with lattice oxygen, being in the Dy^3+^ state. As a result, the size of SnO_2_ microcrystals decreased from 63.1 ± 8.6 nm to 34.9 ± 13.8 nm, and excess tin atoms were released on the surface and were additional centers for chemisorption of oxygen from the air. In this case, the energy band bending *eφ_s_* increased to 0.7–0.65 eV, and the sensor responses to 100 ppm CO grew to *G*_1_/*G*_0_ = 4.7–5.5 compared to the (I)-693K sample (Table 3, Figure 5b). Also, for this sample, the roughness and especially the porosity changed to 5.8 ± 1.3 nm and 30.2 ± 2.2% compared to sample (I)-693 K. In addition, a decrease in the signal intensity in the Raman spectra and a change in the shape of the bands in the region of 100–400 cm^−1^ were observed, which indicates a change in the defectiveness of the film, both before and after the tests (Figure 4, curves (II)-693K and (II)^1^-693K). As a result of the tests under the influence of CO, the response values were significantly reduced (Figure 6b).

In the case of sensors from the (IV)-693K series, the addition of 11.65 at.% Ag impurity in the metallic state affected the Sn–O binding energy and the interaction with 0.4 at.% dysprosium. Due to the lower Dy concentration, the response values were lower than those of sensors from the (II)-693K series (Table 3). With a crystallite size of 31.9 ± 4.4 nm, roughness of 4.6 ± 1.9 nm, and porosity of 27.4 ± 4.8, the intensity and shape of the Raman spectra differed from other samples and changed under the influence of the tests (Figure 4, curves (IV)-693K and (IV)^1^-693K). It is important to note that heat treatment at 723 K of samples ((V)-723K) contributed to a decrease in the crystallite size at high porosity and a further decrease in the response (Figure 6c,d and Table 3).

It is interesting to note that the Raman spectra of all the sensors studied in this work retained weakly expressed bands at 590 and 565 cm^−1^, responsible for the Pt^2+^ and Pd^2+^ ions in the deposited platinum and palladium surfaces, regardless of the film composition and tests under the influence of CO. Apparently, the adsorption of carbon monoxide does not affect the structure of the Pt/Pd bilayer catalyst (Figure 4).

It is assumed a priori that the processes of structural rearrangement of both the films themselves and the deposited catalysts can play a significant role in long-term changes in the properties of thin-film sensors. At the same time, it should be noted that a change in grain size can both improve and worsen the performance characteristics of gas sensors [28]. Therefore, the choice of the optimal grain size should be based on a detailed consideration of all possible consequences of their influence on the parameters of the designed sensors. Diffusion and coagulation phenomena lead to an increase in the size of semiconductor crystallites and a decrease in the degree of dispersion of the catalyst. As a result, the catalyst may be poisoned when exposed to active gases that include CO, which leads to a decrease in the density of adsorption centers of gas molecules. Thus, in order to consider possible mechanisms of degradation of CO sensors based on tin dioxide, it is necessary to analyze all the obtained experimental data together.

Analyzing the experimental results, it can be assumed that the degradation processes of the sensor parameters were due to the fact that in the case of increased porosity and defectiveness of the films, irreversible diffusion and adsorption of CO_2_ molecules in tin dioxide occurred. At the same time, the response values for 10 ppm CO for all the studied sensors were on average *G*_1_/*G*_0_ = 1.13–1.30, as for such low concentrations of gas molecules, the density of adsorption centers on the surface is sufficient. To summarize, it can be concluded that films based on tin oxide doped with antimony (Pt/Pd/SnO_2_: 0.5 at.% Sb) are free from most of the disadvantages found in other samples and show increased stability to carbon monoxide when operating at a constant operating temperature of 673 K.

## 3. Materials and Methods

### 3.1. Sample Preparation

Ceramic targets (CTs) were fabricated by uniaxial static pressing of SnO_2_, Sb_2_O_3_, Dy_2_O_3_, and Ag_2_O powder compacts on a PLG 12 hydraulic press (LabTools, St. Petersburg, Russia) at a pressure of 1500 atm. The targets were sintered in a muffle furnace in an air atmosphere at 1000 °C for 1 h. When obtaining powders for compaction, a chemical method was used based on the co-deposition of metal hydroxides by neutralizing solutions of their salts with an alkaline agent, washing the obtained precipitation of hydroxides, filtering them, drying them, and carrying out thermal destruction. All reagents were of special purity grade. The application of this method to obtain powders of mixed oxides of tin, antimony, silver, and dysprosium is described in [17].

The targets were 75 mm in diameter and 6 mm thick. The 150 µm thick sapphire plates were used as substrates. The deposition of thin films of modified SnO_2_ was carried out by RF magnetron sputtering in an A-500 magnetron (Edwards, Burgess Hill, UK) at a discharge power of 100 W. The working gas contained 56 ± 0.5 vol.% of oxygen, and the rest was argon. The pressure in the chamber was within (6.5–7) 10^−3^ mbar. The target–substrate distance was 80 mm. For 150 nm thick films, the sputtering time was 30 min. Two-layer Pt/Pd catalysts were deposited on the surface of the films by DC magnetron sputtering. First, palladium and then platinum were deposited (each layer was deposited for 15 s). The heater on the back side of the substrate and electrical contacts to the sensitive layers were formed by spraying platinum on the sapphire wafers heated to 773 K with subsequent photolithography engraving before magnetron deposition of the SnO_2_ films. Two photolithography operations were used to form the sensitive elements of a defined shape and size. After the formation of the films, the finished samples were subjected to technological annealing in air at temperatures *T*_an_ of 693 or 723 K for 24 h.

**Figure 7:**
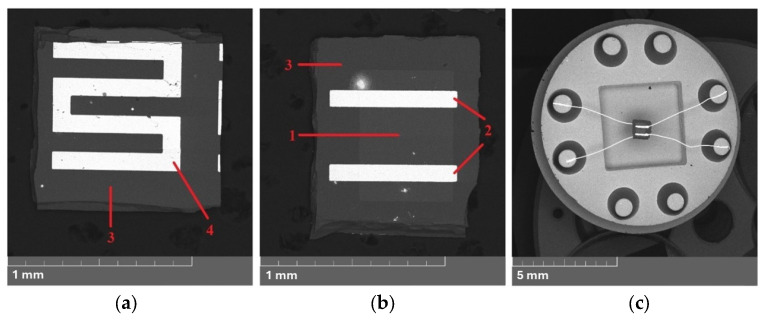
SEM images obtained in the back-scattering (BSE) mode: the sensitive element from the side of (**a**) semiconductor SnO_2_ layer and (**b**) heater; (**c**) sensors assembled into TO-8 case: 1—sensitive element; 2—Pt electrodes; 3—sapphire substrate; 4—Pt heater.

Up to 500 sensors that were 0.7 × 0.7 mm in size with a gas-sensitive film area of 0.3 × 0.3 mm were obtained on a single substrate with a diameter of 30 mm. The wafers were cut into individual parts, after which electrical leads made of gold wire with a diameter of 50 µm were welded to the contact pads of the sensors (using the thermocompression method). The samples were assembled in TO-8 metal cases (Figure 7) [9]. The micromorphology of the surface, chemical compositions, and chemical states of the films were determined using atomic force microscopy (AFM) and X-ray photoelectron spectroscopy (XPS) on specially prepared samples with an area of approximately 10 × 10 mm^2^. Before applying SnO_2_ films of various compositions, a Pt layer was deposited on the sapphire substrate to eliminate the effect of charge accumulation.

We introduced the following numbering of series and designations for films with various additives in the bulk and *T_an_*:

(I)-693K   Pt/Pd/SnO_2_: 0.5 at.% Sb (*T_an_* = 693 K);
(II)-693K  Pt/Pd/SnO_2_: 1 at.% Sb, 1 at.% Dy (*T_an_* = 693 K);
(III)-723K Pt/Pd/SnO_2_: 1 at.% Sb, 1 at.% Dy (*T_an_* = 723 K);
(IV)-693K Pt/Pd/SnO_2_: 1 at.% Sb, 1 at.% Dy, 1 at.% Ag (*T_an_* = 693 K);
(V)-723K  Pt/Pd/SnO_2_: 1 at.% Sb, 1 at.% Dy, 1 at.% Ag (*T_an_* = 723 K).

### 3.2. Sample Characterization

The surface microtopography of the thin films was investigated using atomic force microscopy of the Integra-Aura probe nanolaboratory (NT-MDT, Moscow, Russia) at the research park of St. Petersburg State University (Centre for Diagnostics of Functional Materials for Medicine, Pharmacology, and Nanoelectronics, St. Petersburg, Russia). All measurements were performed in the semi-contact mode using NSG01 probes (Tipsnano, Tallinn, Estonia) with a tip curvature radius of 10 nm, made of single-crystal silicon with a reflective Au coating. Analysis of the nanograin sizes, square roughness, and porosity from AFM scan images was performed by the Gwyddion program ver. 2.50 [29]. The surface porosity was determined as a proportion of the surface area occupied by pores *P = (*Σ*S_i_/S) × 100%*, where Σ*S_i_* is the total surface area of pores and *S* is the scan area.

Survey and core-level (Sn 3*d*, O 1*s*, Sb 3*d*_3/2_, Dy 3*d*, Ag 3*d*, C 1*s*) photoelectron (PE) spectra were measured using an ESCAlab 250 Xi laboratory spectrometer (Thermo Fisher Scientific, Waltham, MA, USA) at the research park of St. Petersburg State University (Centre for Physical Methods of Surface Investigation, St. Petersburg, Russia). A monochromatic AlKα radiation source with *hν* = 1487 eV was used to excite the PE spectra. The chemical state of tin, oxygen, antimony, and silver was analyzed by comparing the shape and binding energies of Sn 3*d*, O 1*s*, Sb 3*d*_3/2_, and Ag 3*d* spectra of the studied samples (I)-693K, (II)-693K, (III)-723K, (IV)-693K, and (V)-723K with the spectra of the reference compounds SnO_2_, antimony pentoxide, and metallic silver Ag^0^. At the same time, the charge state of dysprosium was determined only by comparing the position of the binding energy of the maximum of the corresponding Dy 3*d*_5/2_ line for samples (II)-693K, (III)-723K, (IV)-693K, and (V)-723K with the known literature data for pure compounds Dy^0^ and Dy_2_O_3_ [21,22]. When measuring the survey and core spectra, the analyzer pass energy was 50 eV, and the energy step was 0.1 eV. The position of the maximum of the C 1*s* peak of hydrocarbons on the surface of the samples at 285.0 eV was used as an internal standard for calibrating each spectrum. Detailed analysis of the core-level PE spectra was conducted through peak fitting employing Casa XPS 2.3.16 software [30].

For the characterization of nanosized material and a qualitative probe of the presence of SnO_2_ lattice defects, Raman spectra of samples at room temperature were recorded using a Renishaw InVia Reflex Micro Raman spectrometer (Wotton-under-Edge, UK) with 100 mW power. To improve the signal quality and exclude the appearance of additional bands from the sapphire (Al_2_O_3_) substrate in the Raman spectra of the SnO_2_ films, the laser beam was directed to spots in which there were separating Pt layers acting as contact areas between the gas-sensitive SnO_2_ layer and Al_2_O_3_ support. The Raman spectra of individual sensors of different series were studied before and after long-term tests under the influence of CO. Detailed information on the parameters for measuring spectra by Raman spectroscopy is given in [10].

### 3.3. Sensor Testing Details

**Figure 8:**
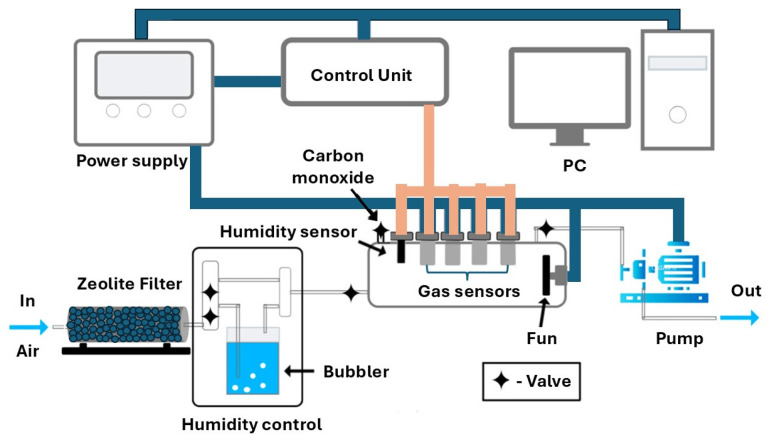
Schematic diagram of the measuring stand.

The time dependences of the film conductivity *G*_0_(t) in clean air, as well as these parameters under the influence of carbon monoxide *G*_1_(t), were measured using a specially designed stand (Figure 8). This stand made it possible to easily reconfigure and stabilize the operating temperature of the sensor, measure the relative humidity in the chamber, and provide operating of sensors in the modes of constant heating and in the thermo-cyclic operation modes. The ratio *G*_1_(T)/*G*_0_(T) was taken as the adsorption response, and the response time *t*_r_ was the stabilization time of 0.9 *G_st_*, where *G_st_* is the stationary value of conductivity. The optimal temperature for measuring the characteristics of CO sensors in the constant heating mode was 673 K, as it provided a sufficient response, and the response time did not exceed 5–10 s. To study the stability of the parameters during long-term operation of sensors exposed to carbon monoxide, measurements of the concentration dependences of the response were carried out in the concentration range of 10–1000 ppm CO every 2–4 days for a period of up to 80–90 days. Based on the time dependences of the conductivity of sensors in the thermal cycling mode in pure air, the values of the energy band bending *eφ_s_* at the grain boundaries in the studied nanocrystalline films were measured using the method developed in [31]. 

To assess the characteristics, four sensors were concurrently positioned in a 1 L quartz chamber that had a fan installed. Two controlled air flows were pumped through the chamber to regulate the humidity level. One flow was dehydrated using zeolite, while the other one was moistened through bubbling. After that, the chamber was sealed. The humidity level was regulated with a Honeywell HIH-4000 (Charlotte, NC, USA) humidity sensor situated in the chamber. The required gas concentration was delivered using a syringe dispenser, which established the needed composition of the gas–air mixture. A balloon containing a mix of 3 vol.% CO in nitrogen was used to introduce carbon monoxide into the measuring chamber. The CO concentration was increased by adding another portion of gas to measure the concentration dependences of the sensor response. Following the measurement, the chamber was filled with clean air at the required humidity level. This paper discusses results from experiments conducted at an average relative humidity of RH = 30%–35%.

## 4. Conclusions

Complex studies of the nanostructure, composition, and electrical and gas-sensitive characteristics of carbon monoxide sensors were conducted based on SnO_2_ thin films obtained by RF magnetron sputtering. Regularities in the change in the size of nanocrystallites, roughness, and porosity of films depending on the composition and temperature of technological annealing were established. The XPS method showed that all films consisted of SnO_2_ and, depending on the sample, contained doping impurities Sb^5+^, Dy^3+^, and Ag^0^. In the Raman spectra in the range of 100–800 cm^−1^, broad bands with a number of maxima associated with size effects were found. During long-term tests under the influence of CO in the range of 10–1000 ppm in the constant heating mode, the Raman spectra for the film of the (I)-693K series (Pt/Pd/SnO_2_: 0.5 at.% Sb) practically did not change, and the sensor parameters remained stable. For sensors of all other series, degradation of parameters was observed during long-term tests, which was associated with a decrease in the size of microcrystals and an increase in porosity and defectiveness of films under the influence of Dy and Ag impurities.

Based on the original SnO_2_ films obtained by high-frequency magnetron sputtering of ceramic targets with an optimal ratio of tin and antimony oxide concentrations, it is possible to manufacture CO sensors based on Pt/Pd/SnO_2_: 0.5 at.% Sb with increased stability at a constant operating temperature of 670 K.

## Figures and Tables

**Table 1:** Statistical data of sample surfaces based on AFM image analysis.

Sample	Root Mean Square Roughness S_q_, nm	Average Grain Size, nm	Surface Porosity, %
(I)-693K	7.4 ± 1.9	63.1 ± 8.6	19.3 ± 2.4
(II)-693K	5.8 ± 1.3	34.9 ± 13.8	30.2 ± 2.2
(III)-723K	9.5 ± 2.2	50.7 ± 15.0	18.7 ± 1.1
(IV)-693K	4.6 ± 1.9	31.9 ± 4.4	27.4 ± 4.8
(V)-723K	8.8 ± 2.4	18.4 ± 4.4	22.2 ± 4.8

**Table 2:** Chemical composition and binding energies of 1*s* and 3*d* electrons of corresponding atoms for thin SnO_2_ films with doping impurities as well as reference compounds according to XPS data.

Sample	Concentration, at.%					Peak Position, eV				
	[Sn]	[O]	[Sb]	[Dy]	[Ag]	Sn3*d*_5/2_	O1*s*	Sb3*d*_3/2_	Dy3*d*_5/2_	Ag3*d*_5/2_
(I)-693K	28.18	71.82	-	-	-	487.0	530.90	-	-	-
(II)-693K	24.66	74.54	0.31	0.49	-	486.85	530.75	540.6	1295.6	-
(III)-723K	25.69	73.39	0.32	0.60	-	486.54	530.44	540.3	1295.6	-
(IV)-693K	22.64	64.95	0.34	0.42	11.65	487.0	530.94	540.8	1295.6	368.4
(V)-723K	25.17	70.1	0.36	0.41	3.95	487.0	530.94	540.8	1295.6	368.4
SnO_2_	32.5	67.5	-	-	-	487.0	530.90	-	-	-
Sb_2_O_5_	-	71.0	29.0	-	-	-		540.6	-	-
Ag^0^	-	-	-	-	100	-	-	-	-	368.2

**Table 3:** Values of resistance *R* measured at an operating temperature of 673 K, energy band bending at grain boundaries *eφ_s_*, and *G*_1_/*G*_0_ responses to the effect of a number of CO concentrations for sensors on films of different compositions before and after testing.

Sample	R, kOm	eφ_s_, eV	G_1_/G_0_ (10 ppm)	G_1_/G_0_ (100 ppm)	G_1_/G_0_ (1000 ppm)
	Before testing				
(I)-693K	10.7	0,23	1.13	2.5	7.13
(II)-693K	3124	0.70	1.32	4.24	17.3
(IV)-693K	9.9	0.35	1.20	2.02	12.7
(V)-723K	7.9	0.25	1.13	1.72	8.7
	After testing under CO exposure for 90 days				
(I)-693K	24	0.23	1.16	2.5	7.64
(II)-693K	20,540	0.53	1.13	1.79	4.24
(IV)-693K	37	0.49	1.22	1.78	3.56
(V)-723K	20	0.41	1.15	1.72	2.75

## Data Availability

The data can be obtained by contacting the correspondence author.
